# Supplementary material for: Cortisol in Manure from Cattle Enclosed with Nofence Virtual Fencing
Source: Animals (Basel). 2022 Nov 3;12(21):3017. doi: 10.3390/ani12213017 (PMC9656181; doi:10.3390/ani12213017)
Supplement: Supplementary file 1 [file animals-12-03017-s001.zip › animals-1969744 - supplementary.pdf]

animals

Article

Cortisol in manure from cattle enclosed with Nofence virtual fencing

Christian Sonne[1,*], Aage Kristian Olsen Alstrup[2,3,*], Cino Pertoldi[4,5], John Frikke[6], Anne Cathrine Linder[4] and Bjarne Styrishave[7]

1 Department of Ecoscience, Aarhus University, Frederiksborgvej 399, DK-4000 Roskilde,
2 Department of Clinical Medicine, Aarhus University, Palle Juul-Jensens Boulevard 165, DK-8200 Aarhus, Denmark
3 Department of Nuclear Medicine and PET, Aarhus University Hospital, Palle Juul-Jensens Boulevard 99, DK-8200 Aarhus, Denmark
4 Department of Chemistry and Bioscience, Section of Bioscience and Engineering, Aalborg University, Fredrik Bajers Vej 7H, DK-9220 Aalborg, Denmark
5 Aalborg Zoo, Mølleparkvej 63, DK-9000 Aalborg, Denmark
6 Wadden Sea National Park, Havnebyvej 30, DK-6792 Rømø, Denmark
7 Toxicology and Drug Metabolism Group, Department of Pharmacy, Faculty of Health and Medical Sciences, University of Copenhagen, Universitetsparken 2, DK-2100, Denmark
* Correspondence: Aage Kristian Olsen Alstrup aagealst@rm.dk (AKOA); cs@ecos.au.dk (CS)

Table S1. Information on the animals including hormones and warning of the 5 cows included in the present study. The cumulative values also include shocks and warnings received on days between the collection of manure samples. Note that manure samples on 29-05-2021 were collected prior to the virtual boundary being introduced later that day.

Cow ID	Born	Date	Fence type	Sample name	Sample number	CORT (ng/g ww)	E2 (ng/g ww)	Prog (ng/g ww)	Shocks (*n*)	Cumulative shocks (*n*)	Warnings (*n*)	Cumulative warnings (*n*)	Cumulative shocks and warnings (*n*)
35-5	26-03-2016	29-05-2021	Traditional	Manure 2	35-5-1	23.0	22.1	40.5					
			Traditional + Virtual						3	3	6	6	9
		31-05-2021	Traditional + Virtual	Manure 25	35-5-2	20.2	21.1	21.4	0	3	0	7	10
		01-06-2021	Traditional + Virtual	Manure 26	35-5-3	20.3	19.8	43.4	0	3	0	7	10
		03-06-2021	Traditional + Virtual	Manure 27	35-5-4	16.5	23.8	18.9	0	3	0	7	10
		05-06-2021	Traditional + Virtual	Manure 28	35-5-5	15.5	18.6	24.8	0	4	5	13	17
		07-06-2021	Traditional + Virtual	Manure 29	35-5-6	20.2	19.3	60.5	0	4	1	23	27
		09-06-2021	Traditional + Virtual	Manure 30	35-5-7	17.7	25.6	75.9	0	4	2	29	33
		11-06-2021	Traditional + Virtual	Manure 31	35-5-8	24.3	23.7	16.4	0	8	10	46	54

Copyright: © 2022 by the authors. Licensee MDPI, Basel, Switzerland. This article is an open access article distributed under the terms and conditions of the Creative Commons Attribution (CC BY) license (https://creativecommons.org/licenses/by/4.0/).

		13-06-2021	Virtual	Manure 32	35-5-9	16.3	25.1	25.9	0	8	4	50	58
		15-06-2021	Virtual	Manure 1	35-5-0	41.9	40.1	175.6	0	8	1	51	59
39-2	28-03-2016	29-05-2021	Traditional	Manure 33	39-2-5	16.1	31.5	37.3					
			Traditional + Virtual (PM)						3	3	6	6	9
		31-05-2021	Traditional + Virtual	Manure 7	39-2-4	20.4	22.6	110.7	0	3	0	6	9
		01-06-2021	Traditional + Virtual	Manure 4	39-2-1	19.9	28.9	60.2	0	3	2	8	11
		03-06-2021	Traditional + Virtual	Manure 9	39-2-7	16.2	28.4	54.1	0	4	0	13	17
		05-06-2021	Traditional + Virtual	Manure 5	39-2-2	25.5	30.2	47.7	1	5	9	31	36
		07-06-2021	Traditional + Virtual	Manure 6	39-2-3	16.3	36.2	37.2	0	5	0	33	38
		09-06-2021	Traditional + Virtual	Manure 34	39-2-8	24.4	21.6	41.3	0	5	0	33	38
		11-06-2021	Traditional + Virtual	Manure 3	39-2-0	23.0	29.3	33.4	0	8	3	40	48
		13-06-2021	Virtual	Manure 8	39-2-6	22.3	44.7	44.6	0	8	2	42	50

		15-06-2021	Virtual	Manure 35	39-2-9	19.2	19.6	43.7	0	8	0	42	50
		29-05-2021	Traditional	Manure 15	40-3-6	27.6	22.8	33.8	3	3	3	3	6
		31-05-2021	Traditional + Virtual	Manure 14	40-3-5	21.8	26.0	29.5	0	3	1	4	7
		01-06-2021	Traditional + Virtual	Manure 11	40-3-1	22.3	22.7	40.1	0	3	0	4	7
		03-06-2021	Traditional + Virtual	Manure 36	40-3-4	17.0	18.9	27.2	1	4	3	10	14
40-3	05-10-2016	05-06-2021	Traditional + Virtual	Manure 37	40-3-7	21.9	18.2	36.0	1	5	12	25	30
		07-06-2021	Traditional + Virtual	Manure 38	40-3-8	15.6	14.7	18.3	0	5	0	26	31
		09-06-2021	Traditional + Virtual	Manure 12	40-3-2	31.7	42.9	77.0	0	5	0	27	32
		11-06-2021	Traditional + Virtual	Manure 13	40-3-3	17.6	41.8	25.0	0	8	4	36	44
		13-06-2021	Virtual	Manure 10	40-3-0	27.1	37.1	101.9	0	8	1	39	47
		15-06-2021	Virtual	Manure 39	40-3-9	14.1	16.4	20.6	0	8	2	43	51
2005-1	16-05-2012	29-05-2021	Traditional	Manure 16	2005-1-0	26.9	31.2	33.6					

			Traditional + Virtual (PM)						3	3	4	4	7
		31-05-2021	Traditional + Virtual	Manure 17	2005-1-1	22.9	41.1	78.8	0	3	3	8	11
		01-06-2021	Traditional + Virtual	Manure 40	2005-1-4	16.9	20.6	28.7	0	3	8	16	19
		05-06-2021	Traditional + Virtual	Manure 18	2005-1-2	20.0	27.8	40.3	0	4	18	40	44
		07-06-2021	Traditional + Virtual	Manure 41	2005-1-5	25.2	21.9	32.9	0	5	7	60	65
		09-06-2021	Traditional + Virtual	Manure 42	2005-1-6	18.9	20.3	47.2	0	6	3	70	76
		11-06-2021	Traditional + Virtual	Manure 19	2005-1-3	21.5	21.2	64.1	0	9	1	79	88
		13-06-2021	Virtual	Manure 43	2005-1-7	25.2	21.4	35.6	0	9	3	82	91
		15-06-2021	Virtual	Manure 44	2005-1-8	14.1	17.7	23.9	0	9	4	86	95
		29-05-2021	Traditional	Manure 45	2281-4-3	21.6	19.6	55.1					
2281-4	07-02-2016		Traditional + Virtual (PM)						3	3	4	4	7
		31-05-2021	Traditional + Virtual	Manure 46	2281-4-4	18.0	14.9	28.9	0	3	5	9	12

01-06-2021	Traditional + Virtual	Manure 23	2281-4-6	14.5	24.0	31.2	1	4	6	15	19
03-06-2021	Traditional + Virtual	Manure 47	2281-4-8	19.1	15.6	26.2	0	4	0	15	19
05-06-2021	Traditional + Virtual	Manure 22	2281-4-5	16.9	21.3	34.7	0	4	9	28	32
07-06-2021	Traditional + Virtual	Manure 20	2281-4-1	24.1	23.2	23.4	0	5	7	39	44
09-06-2021	Traditional + Virtual	Manure 48	2281-4-9	13.3	13.9	44.1	0	6	0	62	68
11-06-2021	Traditional + Virtual	Manure 24	2281-4-7	16.5	14.8	24.4	0	10	2	74	84
13-06-2021	Virtual	Manure 49	2281-4-10	23.0	25.0	59.6	0	10	1	75	85
15-06-2021	Virtual	Manure 21	2281-4-2	12.8	17.6	19.4	0	10	2	77	87

Statistical analyses

We considered using mixed models though it violate the assumption of repeated measures tests being the sphericity. This imply equal variances of the differences between all combinations of groups as seen from the cortisol figures. Unfortunately, it has not been possible to apply a mixed model being equivalent to the parametric ANOVA with repeated measurements (blocked ANOVA) and the non-parametric Friedmann's test. The last we have also considered given the fact that the amount of repeated measurements is small, with consequences for the normality of the data etc. Therefore, we preferred to pool the samples being aware that the cortisol values of the excrements laid by the single cattle are not independent from each other, and also being aware that also the cattle interact with each other and therefore the cortisol values is probably also not independent among individuals. We show here the correlation matrix of the cortisol level among individuals;

	Cow 2	Cow 3	Cow 4	Cow 5
Cow 1				
Cow 2	-0,2191			
Cow 3	0,43604	0,086075		
Cow 4	-0,36113	-0,62088	0,096658	
Cow 5	-0,54708	0,65799	-0,22575	-0,39089

It is seen that there are several cases with strong positive or negative correlations between individuals. Because of this "dependency" of the data, we decided also to analyse the cortisol trend in every single cattle considered individually and also in this case we found the same results (no trends) that we have found by pooling the sample.

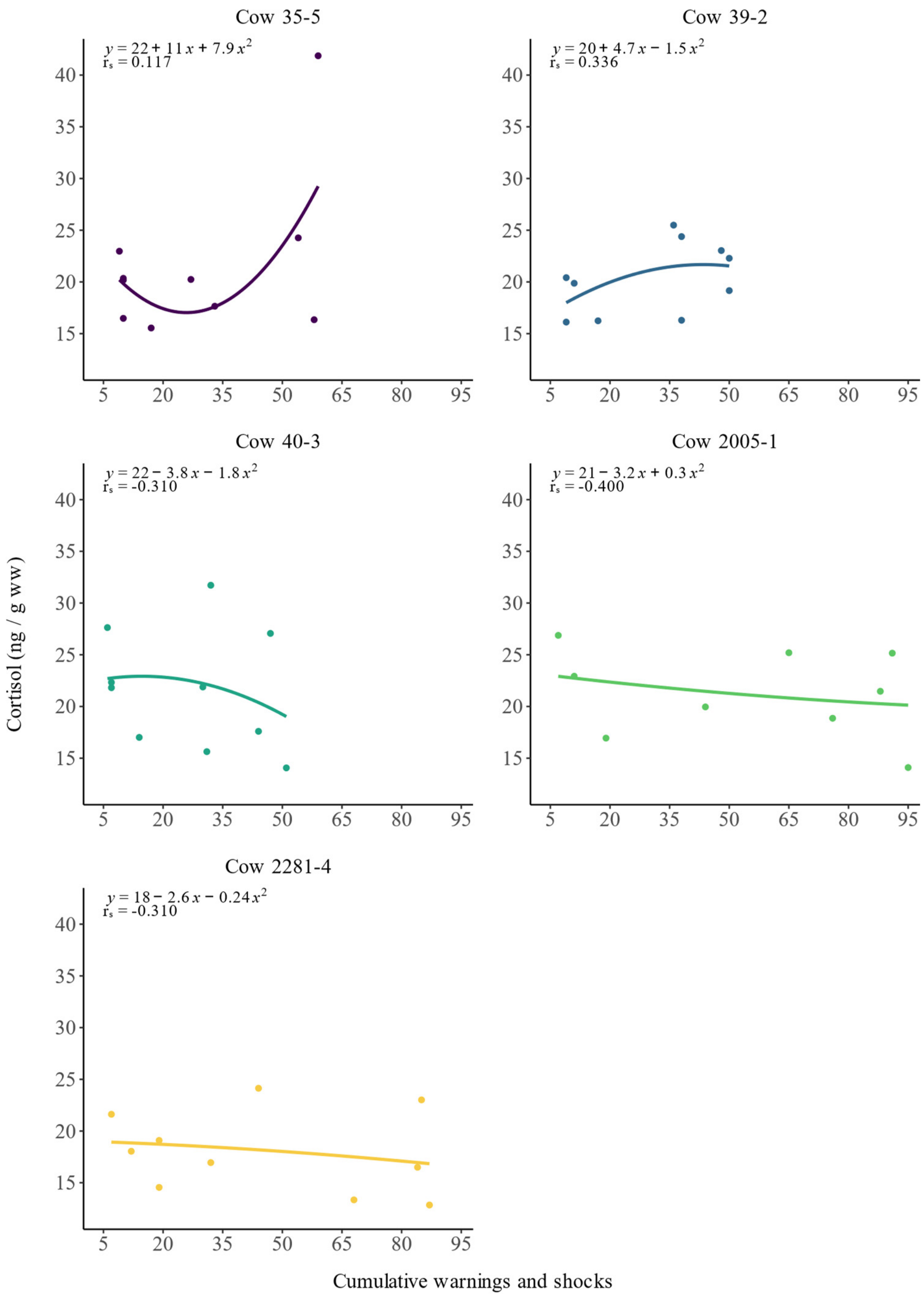

Figure S1. Polynomial (second degree) regression of cortisol concentration in relation to the cumulative number of auditory warnings and electric impulses received for each individual during the study period May 29 to June 15. The regression equation and the spearman correlation coefficient (r_s) are given in each plot.

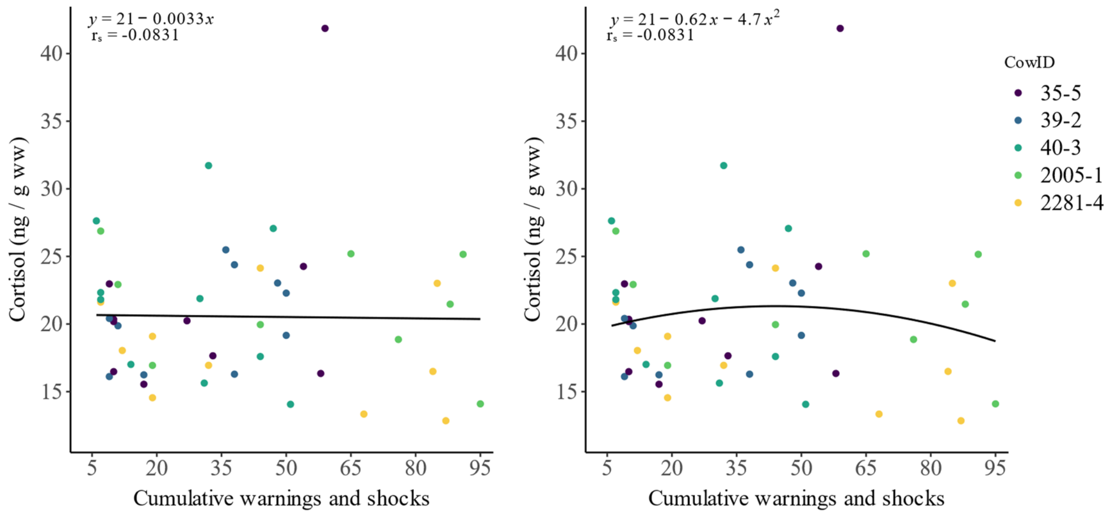

Figure S2. Linear (left) and polynomial (right) regression of cortisol concentration in relation to the cumulative number of auditory warnings and electric impulses received during the study period May 29 to June 15. The regression equation and the spearman correlation coefficient (r_s) are given in each plot.
